# COPD and asthma in patients with opioid dependency: a cross-sectional study in primary care

**DOI:** 10.1038/s41533-019-0161-7

**Published:** 2020-01-14

**Authors:** S. Mehta, N. Parmar, M. Kelleher, C. J. Jolley, P. White, S. Durbaba, M. Ashworth

**Affiliations:** 1 School of Population Health and Environmental Sciences, King’s College London, Guy’s Campus, Addison House, London, SE1 1UL UK; 2 South London & Maudsley NHS Foundation Trust, Lorraine Hewitt House, 12-14 Brighton Terrace, Brixton, SW9 8DG UK; 3 Centre for Human & Applied Physiological Sciences, School of Basic & Medical Biosciences, Faculty of Life Sciences & Medicine, King’s College London, Shepherd’s House, Rm 4.4, Guy’s Campus, London, SE1 1UL UK

**Keywords:** Drug regulation, Health policy, Outcomes research

## Abstract

Patients treated for drug addiction have high asthma and COPD prevalence rates. The relative contributions of cigarette smoking, smoking intensity and possible smoking of other substances has not been described. We aimed to describe the prevalence and determinants of asthma and COPD in patients prescribed methadone as opioid substitution therapy (OST). In a cross-sectional study of an anonymised patient-level primary care dataset of UK inner-city general practices (*n* = 46), 321,395 patients aged ≥18 years were identified. A total of 676 (0.21%) had a record of a methadone ever issued in primary care. The association between respiratory disease and methadone prescribing was examined using logistic regression. Models were adjusted for potential effects of clustering by practice. A total of 97.3% of patients prescribed methadone were cigarette smokers, either current (81.2%) or ex-smokers (16.1%). The prevalences of asthma and COPD were higher in methadone patients (14.2% and 12.4%, respectively) compared to non-methadone patients (4.4% and 1.1%, respectively). Methadone was an independent determinant of asthma, adjusting for smoking status (OR 3.21; 95% CI: 2.52, 4.10) or for smoking intensity (3.08; 2.27, 4.19), and of COPD, adjusting for smoking status (6.00; 4.61, 7.80) or for smoking intensity (5.80; 4.12, 8.17). COPD and asthma prevalence were substantially higher in those prescribed methadone compared to those never prescribed methadone. Prescription of methadone was an independent predictor for both COPD and asthma, even after adjustment for smoking status and smoking intensity. Possible explanations include confounding by association with smoking of heroin or crack cocaine, both of which may have a causal association with COPD and asthma.

## Introduction

Opioid dependency is the most common addiction among patients seeking treatment for substance misuse.^1^ In England, of almost 300,000 adults in contact with structured addiction treatment programmes, just over half were receiving treatment for opioid dependency with ~20% treated in primary care.^1,2^ Injecting drug users accounted for almost half of patients entering drug treatment.^1,3^ Since 2003, the proportion of heroin users aged over 40 years entering treatment has trebled, constituting an ageing cohort effect.^4^

Substitution therapy with opioid agonists is the mainstay of treatment for patients addicted to opiates. Randomised controlled trials provide evidence of the effectiveness of opioid substitution therapy (OST), such as methadone replacement, in healthcare settings.^5,6^ Treatment benefits include harm reduction related to drug-seeking behaviour, reduced rates of blood-borne infections and fatal overdose.^7–9^ OST increases the overall time to heroin relapse and maximum consecutive days of abstinence when compared with placebo.^7^

Despite the availability of treatment programmes, mortality rates related to opioid addiction are high, particularly in those who inject drugs or are older.^10–12^ In a study of opiate users in London, the standardised mortality rate (SMR) was 12 times that of the general population; 43% of deaths in opiate users were attributable to drug-related poisoning.^11,13^ Higher death rates were attributed to risk-related behaviours, co-morbidity, smoking and deprivation. Mortality in injecting users was found to be attributable to skin and groin infections, blood-borne virus infection, deep vein thrombosis (DVT) and pulmonary emboli.^14^ Also reported was an SMR of 8.9 for respiratory disease.^14^

High prevalence rates of asthma and chronic obstructive pulmonary disease (COPD) have been reported in patients receiving treatment for substance misuse.^15^ Evidence suggests that the method of substance misuse is important, with respiratory health poorer in those that smoke heroin.^16,17^ One recent cross-sectional study of substance misuse by inhalation noted a high prevalence of COPD (35%) and asthma–COPD overlap syndrome (15%).^18^ Asthma appears to be poorly managed in these patients among whom asthma deaths have been linked to substance misuse.^15,19–21^ There are several mechanisms by which opioids may contribute to the cause or consequences of respiratory disease, including suppression of neural respiratory drive, increased airway resistance and as an irritant stimulating histamine release.^22,23^

Underlying respiratory disease exacerbates the respiratory complications of opiate use and leads to higher risk of death.^22–24^ Opioids, such as methadone, are associated with respiratory depression and even respiratory arrest, particularly on treatment induction.^25^ Tobacco smoking is the most important cause of COPD and impairs asthma control. High smoking rates in patients receiving OST have been reported, although previous studies have not reported cigarette smoking intensity (number of cigarettes smoked per day) in this group, which may have resulted in an underestimate of the role of heavy smoking.^15,26^ We aimed to describe the prevalence and determinants of asthma and COPD in patients receiving OST with particular focus on the role of smoking intensity.

## Results

A total of 321,395 patients aged ≥18 years were identified in Lambeth DataNet (LDN). Of these, 676 (0.21%) had a record of a methadone prescription issued in primary care. Of the 46 practices providing data, five practices did not have any registered patients with a record of a methadone prescription in primary care.

The characteristics of patients in the methadone and non-methadone groups, as well as values for smoking status and smoking intensity are summarised in Table 1.

**Table 1:** The characteristics of patients with or without a record of methadone prescription in primary care.

	Methadone group (*n* = 676)	Non-methadone group (*n* = 320,719)	*p*-value for difference*
Gender			
Male	70.7%	50.4%	*p* < 0.001
Age			
(mean, in years)	48.9	41.5	*p* < 0.001
Ethnicity			
South Asian	2.9%	7.8%	*p* < 0.001
Black African	8.9%	21.4%	*p* < 0.001
Mixed	5.3%	4.9%	*p* < 0.001
Other	2.7%	3.2%	*p* < 0.001
White	80.2%	62.6%	*p* < 0.001
IMD-2015 Score	29.5	28.8	*p* < 0.001
Smoking status			
Current smoker	81.2%	20.6%	*p* < 0.001
Ex-smoker	16.1%	20.3%	*p* < 0.01
Never smoked	2.7%	59.1%	*p* < 0.001
Smoking intensity			
≥1 to <10 cigarettes	34.5%	52.9%	*p* < 0.001
≥10 to <20 cigarettes	44.0%	34.1%	
≥20 to <40 cigarettes	19.7%	12.2%	
≥40 cigarettes	1.8%	0.9%	
Alcohol consumption			
(% non-alcohol drinkers)	41.3%	24.3%	*p* < 0.001

*p-Values obtained from *t*-test for comparison of continuous variables (age and IMD-2015 values) and from Pearson’s *χ*^2^ test for comparison of categorical variables (all other values)

Smoking status data were available for 94.9% of the total sample. Almost all (97.3%) those prescribed methadone were smokers or ex-smokers, in contrast to the non-methadone group in which smokers or ex-smokers comprised 40.9%. Smoking intensity data (cigarettes per day) were recorded for 67.5% of the sample (67.6% of the non-methadone group and 53.3% of the methadone group).

The prevalence of COPD, asthma and both conditions concurrently in the methadone and non-methadone groups are summarised in Table 2. Multivariable analysis produced values for the adjusted odds ratios (ORs) of COPD or asthma according to smoking status, smoking intensity and prescription of methadone (Tables 3a, b and 4a, b). For COPD, current smoking was the strongest determinant, OR 23.47 (95% confidence intervals: 20.47, 26.92). Smoking status remained a strong determinant of COPD in ‘ex-smokers’ (see Table 3a, b). Smoking intensity was also associated with COPD with stronger associations for higher smoking intensity categories; the adjusted OR for those smoking ≥40 cigarettes/day was 35.26 (25.73, 48.30).

**Table 2:** Prevalence of COPD and asthma in the methadone and non-methadone groups.

	Methadone group (*n* = 676)	Non-methadone group (*n* = 320,719)	Pearson’s *χ*^2^, *p*-value
COPD (%)	12.4%	1.1%	*p* < 0.001
Asthma (%)	14.2%	4.4%	*p* < 0.001
COPD and asthma*	5.0%	0.3%	*p* < 0.001

*Patients with an electronic record of both diagnoses

**Table 3:** **a** The adjusted risk of COPD according to methadone prescription and smoking status. **b** The adjusted risk of COPD according to methadone prescription and smoking intensity.

	Adjusted odds ratios, with 95% confidence intervals*
**a**	
Methadone	6.00 (4.61 to 7.80)
Smoking status: current smoker	23.47 (20.47 to 26.92)
Smoking status: ex-smoker	11.74 (10.31 to 13.37)
Age	1.11 (1.10 to 1.12)
Gender	1.06 (0.98 to 1.15)
IMD-2015 score	1.04 (1.03 to 1.05)
South Asian ethnicity	0.75 (0.63 to 0.88)
Black ethnicity	0.44 (0.39 to 0.49)
**b**	
Methadone	5.80 (4.12 to 8.17)
Smoking intensity: <10 cigarettes/day	5.57 (5.04 to 6.17)
Smoking intensity: 10 to <20 cigarettes/day	11.41 (10.39 to 12.53)
Smoking intensity: 20 to <40 cigarettes/day	20.36 (18.17 to 22.82)
Smoking intensity: ≥40 cigarettes/day	35.26 (25.73 to 48.30)
Age	1.10 (1.097 to 1.103)
Gender	1.46 (1.35 to 1.58)
IMD-2015 score	1.04 (1.03 to 1.05)
South Asian ethnicity	0.51 (0.43 to 0.61)
Black ethnicity	0.35 (0.32 to 0.40)

*Reference groups for categorical data:

smoking status: smokers and ex-smokers compared with never smokers;

gender: women compared with men;

ethnic groups: each compared with White ethnic group.

*Reference groups for categorical data:

smoking intensity: smoking intensity categories compared with never smokers;

gender: women compared with men;

ethnic groups: each compared with White ethnic group

**Table 4:** **a** The adjusted risk of asthma according to methadone prescription and smoking status. **b** The adjusted risk of asthma according to methadone prescription and smoking intensity.

	Adjusted odds ratio, with 95% confidence intervals*
**a**	
Methadone	3.21 (2.52 to 4.10)
Smoking status: current smoker	1.05 (0.999 to 1.104)
Smoking status: ex-smoker	1.39 (1.33 to 1.45)
Age	1.02 (1.015 to 1.017)
Gender	0.74 (0.72 to 0.77)
IMD-2015 score	1.01 (1.002 to 1.011)
South Asian ethnicity	1.22 (1.14 to 1.30)
Black ethnicity	1.09 (1.04 to 1.14)
**b**	
Methadone	3.08 (2.27 to 4.19)
Smoking intensity: <10 cigarettes/day	1.42 (1.32 to 1.52)
Smoking intensity: 10 to <20 cigarettes/day	1.53 (1.41 to 1.66)
Smoking intensity: 20 to <40 cigarettes/day	1.49 (1.31 to 1.69)
Smoking intensity: ≥40 cigarettes/day	1.21 (0.74 to 1.99)
Age	1.02 (1.015 to 1.018)
Gender	0.79 (0.76 to 0.82)
IMD-2015 score	1.01 (1.001 to 1.010)
South Asian ethnicity	1.16 (1.09 to 1.24)
Black ethnicity	1.02 (0.97 to 1.06)

*Reference groups for categorical data:

smoking status: smokers and ex-smokers compared with never smokers;

gender: women compared with men;

ethnic groups: each compared with White ethnic group

*Reference groups for categorical data:

smoking intensity: smoking intensity categories compared with never smokers;

gender: women compared with men;

ethnic groups: each compared with White ethnic group

For asthma, the association with smoking in each model was either not significant or less marked. Current smoking was not an independent determinant although ex-smokers were more likely to have asthma, OR 1.39 (1.33, 1.45) (see Table 4a).

Methadone prescription was an independent determinant of COPD and asthma in all models. For COPD, the adjusted OR for methadone was 6.00 (4.61, 7.80), after adjustment for smoking status. Further adjustment for smoking intensity resulted in an OR of 5.80 (4.12, 8.17) for methadone prescription in current smokers (Table 3a, b). Similarly, for asthma, methadone prescription was an independent determinant: OR 3.21 (2.52, 4.10) after adjustment for smoking status and OR 3.08 (2.27, 4.19) following adjustment for smoking intensity (Table 4a, b).

## Discussion

This is the first study to show that COPD and asthma prevalence are substantially higher in those prescribed methadone compared to those never prescribed methadone, even after adjustment for high smoking rates and smoking intensity. Almost all those in the methadone group were either smokers or ex-smokers. Based on regression modelling, we were able to confirm known associations between COPD, smoking status and smoking intensity.^27^ However, cigarette smoking did not appear to be the sole factor accounting for high COPD prevalence rates in the methadone group. Prescription of methadone itself was an independent predictor for both COPD and asthma.

Possible explanations for the association between methadone prescription and respiratory disease include confounding by association with smoking of heroin or crack cocaine, both of which are associated with COPD and asthma.^16,17^ Concurrent cannabis inhalation may also contribute to respiratory symptoms although detailed spirometry and plethysmography studies have found little evidence that cannabis causes airways obstruction.^28,29^ It is not plausible that consumption of prescribed oral methadone itself has a causal relationship with respiratory disease although respiratory depression may contribute both to symptom presentation or symptom masking.

A causal relationship between heroin smoking and early onset COPD, with predominant emphysema phenotype, has been postulated.^17^ One study found that 95% of patients who smoke crack have respiratory symptoms and 65% of patients find these symptoms bothersome.^30^ Persistent respiratory symptoms may be attributable to the development of hypersensitivity of pulmonary epithelial cells, triggered following toxic damage by inhalation of heroin and co-contaminants.^31,32^

Previous studies have reported the onset of wheeze and chest tightness, with a temporal relationship to inhalation of heroin.^32^ Several physiological mechanisms have been postulated for heroin-induced asthma. Nasal lavage with saline showed the presence of basophils and eosinophils, suggesting an allergic mechanism which causes bronchospasm.^32^ Other explanations, include a direct pharmacological mechanism which stimulates mast cell degranulation and a histamine response.^32^ Heroin is known to trigger reversible bronchoconstriction through histamine release triggering acute onset asthma.^33^ Both cocaine and heroin when inhaled may trigger respiratory disease through direct thermal injury; cocaine, cannabis and associated contaminants are known to have a direct inflammatory effect on the respiratory epithelium.^17,34^ These studies support the importance of screening emergency presentations of asthma for substance misuse and specifically by inhalation.^32,33^

A particular strength of this study was the large primary care dataset with high recording levels of smoking data. The use of primary care records enables comparison between methadone and non-methadone users. Recording of long-term conditions such as COPD and asthma is likely to be more comprehensive in primary care records than in the secondary care records of addiction services. The recording of COPD and asthma status, and diagnostic criteria of both, are incentivised in primary care and should maximise the availability of coded data using standard Read codes.^35^

Nevertheless, we were unable to explore disease severity, prescribing of guideline-recommended treatment, symptom control or medication adherence with respect to either COPD or asthma. It is likely that the recorded prevalence of both COPD and asthma were underestimates of true prevalence.^36,37^ Respiratory depression following opiate use, even in opiate-tolerant patients, may have masked respiratory symptoms and contributed to under-recognition of underlying respiratory disease.^22,38^ Coded data on behavioural factors such as self-care and social support were not available and these factors, combined with access difficulties, are likely to have contributed to disease severity and treatment delays and may also have contributed to under-reporting of prevalence.^39,40^ COPD disease severity may be estimated from primary care records using annual Quality and Outcomes Framework (QOF) recording of spirometry findings. However, in a post-hoc analysis of patients with COPD, only 8.1% in the methadone group and 75.9% in the non-methadone group had a valid spirometry record within the previous year. Our findings could not therefore be linked to severity of respiratory disease in those prescribed methadone.

We were able to adjust our analysis for age and smoking intensity, but we could not adjust for possible confounders such as the pack-years of smoking or method of smoking, for example depth of inhalation or smoking down to the cigarette filter. We were unable to adjust for secondary smoke exposure, which may be high in substance misuse patients and associated with shared accommodation and socialising with other smokers.^41^ Methadone is occasionally prescribed in palliative care, and we were unable to account for this indication for methadone prescription.^42^ General practice case-notes rarely contain coded data of inhaled heroin or cocaine use although such information would have supported our assumptions about possible causality.^17,38^

Our study captured data for patients prescribed methadone in primary care and did not account for those prescribed methadone in local specialist addiction services. National estimates indicate that only 21% of methadone prescribing takes place in primary care, with the majority prescribed by specialist community services.^1^ The area of study was atypical in that approximately 51% of all methadone prescribing took place in primary care in the study year (personal communication, MK, October 2017). Identifying patients in primary care with opioid dependency but treated in specialist clinics is problematic. These patients may not attend primary care, and if they do, their dependency may not be coded. In a post-hoc analysis, a search for opioid dependency codes without associated methadone prescribing codes only identified a further 61 (9.0%) patients, implying substantial under-recording of opioid dependency among patients treated outside primary care. Patients managed by specialist services are likely to have greater severity of both addiction problems and associated COPD with one secondary care study of a selected population reporting a COPD prevalence rate of 36.4%.^43^ The cohort of primary care patients included in our study may have included some patients seen predominantly by specialist community services but prescribed one or more emergency methadone prescriptions in primary care. Nevertheless, a study based in primary care may inaccurately estimate the prevalence of respiratory comorbidity in this group if only 50% of the target population is included.

Our findings build on previous evidence linking opioid dependency with respiratory disease. Previous studies have reported the link between acute asthma and mortality related to substance misuse.^38,44,45^ Increased risk of asthma and COPD was found in a large study of drug users in Scotland, but the sample included all ‘drug misuse’ and did not specify the relationship between respiratory disease and methadone prescription; smoking intensity was also not reported.

Further work is needed to identify the relative contribution of cigarette smoking, heroin and cocaine inhalation to the recorded prevalence of COPD and asthma and to the severity of both conditions. Given the high prevalence of respiratory co-morbidity, further studies should explore safety aspects of primary care management, including the potential for inhaler devices to be used to inhale other volatile substances.^46^

Our findings have emphasised the importance of the physical health of substance misuse patients. Current configuration of addiction services in the UK has resulted in the separation of addiction management from the management of long-term conditions. Separate commissioning of specialist addiction services may act as a barrier for integrated approaches to health.^47^ Smoking is a key public health concern in this cohort which highlights the importance of ensuring access to smoking cessation services.^48–50^

Our findings may have provided an underestimate of the prevalence of respiratory disease in patients prescribed methadone. It is possible that patients treated in secondary care have more severe addiction, higher co-morbidity prevalence and less effective long-term condition management. The development of more integrated pathways of care, with access to spirometry, smoking cessation services, and specific disease management for COPD and asthma appears to be a priority for patients on OST.^51,52^

The respiratory depressant effect of methadone may contribute to mortality rates in methadone users. Further guidance is needed on opiate prescribing in patients with obstructive airway disease.^25,53^

In summary, patients on methadone replacement have high prevalence of both COPD and asthma. Smoking rates are also high in these patients. The independent association between methadone prescription and respiratory disease may be mediated by the smoking of other substances in addition to being a direct effect of methadone. The high burden of respiratory disease highlights the importance of generalist care and supports the co-commissioning and integration of services for these patients.

## Methods

### Study design

We conducted a cross-sectional study using patient-level data derived from LDN, a database of primary care data extracted from the electronic patient records of general practices in the London Borough of Lambeth. At the time of this study, the database contained anonymised case records of 386,516 patients registered at all general practices in Lambeth (*n* = 46) and stored in accordance with UK NHS information governance requirements. Ethical approval for the analysis of patient data which is fully pseudonymised and pseudonymised at source is not required (confirmation that LDN fulfils these requirements received from Health Research Authority, 15/2/17). This study was approved by the Lambeth DataNet Steering Group and received proportionate review by the Healthcare Research Authority.

### Study measurements

We extracted data from all patients aged 18 years and over and identified all those with a record of ever having been prescribed methadone in primary care. Data were extracted in February 2017. We obtained demographic data on all registered patients covering age, gender, ethnicity, social deprivation; also smoking and alcohol consumption. Social deprivation was defined by the Index of Multiple Deprivation (IMD) 2015 score and mapped to lower super-output area, the smallest area for which data are available, in order to prevent accidental identification of participants.^54^ Smoking status was categorised as current smoker, ex-smoker or never smoker. Smoking intensity data consisted of a record of the number of cigarettes smoked per day. Alcohol consumption was recorded as the number of units consumed per week. Asthma and COPD disease clinical codes (Read codes) were extracted; identification of patients with asthma and COPD was based on validated QOF codes.^55^

### Exclusion criteria

Patients were excluded from the analysis if they were under 18 years of age. We did not include patients prescribed other opioid agonists such as buprenorphine since we were unable to distinguish whether this was prescribed for addiction therapy or as analgesia.

### Statistical analysis

The demographic characteristics of patients prescribed methadone (the ‘methadone group’) and those not prescribed methadone (the ‘non-methadone group’) were compared using univariate analysis. Mean values for both groups were obtained for demographic variables, smoking and alcohol data (‘lifestyle data’) and the prevalence of asthma and COPD. Proportions were compared using *χ*^2^ tests of significance and *p* values obtained from *t*-test for comparison of continuous variables.

We explored the association between the diagnosis of COPD or asthma, and methadone prescribing using multivariable logistic regression, adjusting for smoking, age, gender and deprivation. Models were also adjusted for potential clustering effects of clustering by practice. In these models, either COPD or asthma was entered as the outcome variable; binary predictor variables were classified as categorical variables. Separate models were constructed to examine the impact of smoking, using smoking status in the first and smoking intensity in the second instance. Never-smokers were the comparator in each model.

Primary care records do not contain data enabling us to identify the total duration of smoking (‘pack-years’). If we confined our analysis to ‘current smokers’, we would have been unable to interpret the role of previous smoking. We therefore analysed the role of smoking as a determinant of COPD or asthma in two ways: ‘current smoker’ and ‘ex-smoker’. Each definition of smoking status was used to conduct a separate regression model. Data regarding mode of substance misuse, for example inhalation or injecting was not coded in primary care data.

LDN contains data on smoking intensity (for ‘current smokers’ and ‘ex-smokers’), which were used to refine the regression models. Smoking intensity was categorised into: ‘1 to <10 cigarettes/day’, 10 to <20 cigarettes/day’, ‘20 to <40 cigarettes/day’ and ‘≥40 cigarettes/day’.

All analyses were conducted using Stata software version 15.0 (StataCorp LP, College Station, TX, USA) analysis.

### Reporting Summary

Further information on research design is available in the Nature Research Reporting Summary linked to this article.

## Supplementary information

Reporting Summary

## Data Availability

The data that support the findings of this study are available from Lambeth DataNet (https://www.lambethccg.nhs.uk/your-health/Information-for-patients/lambeth-datanet/Pages/default.aspx). Restrictions apply to the availability of these data which were used under license for the current study. We do not have permission to give public access to these data.
